# Dynamic Changes and Characterization of the Metal Ions in the Silk Glands and Silk Fibers of Silkworm

**DOI:** 10.3390/ijms24076556

**Published:** 2023-03-31

**Authors:** Qingsong Liu, Xin Wang, Yifan Zhou, Xiaoyin Tan, Xiaoqian Xie, Yi Li, Haonan Dong, Zhangchen Tang, Ping Zhao, Qingyou Xia

**Affiliations:** 1 Integrative Science Center of Germplasm Creation in Western China (Chongqing) Science City, Biological Science Research Center, Southwest University, Chongqing 400715, China; 2 Chongqing Engineering and Technology Research Center for Novel Silk Materials, Chongqing Key Laboratory of Sericulture, Southwest University, Chongqing 400715, China; 3 Sericulture Genome and Biotechnology Engineering Laboratory, Chongqing 400715, China

**Keywords:** silk gland, silk fiber, metal elements

## Abstract

Metal ions are involved in the conformational transition of silk fibroin and influence the structure and mechanical properties of silk fibers. However, the dynamic characteristics of metal ions during the formation of silk fibers remain unclear. In this study, we found that the silk glands of silkworms contain various metal elements, with varying levels of the metal elements in different zones of the glands and higher levels in the anterior silk glands. Additionally, the content of various metallic elements in the silk glands varied greatly before and after spinning, similar to their content in different cocoon layers, thus, indicating that the anterior silk glands maintain a certain metal ion environment for the transport and conformational transformation of the silk proteins. Most of the metallic elements located in fibroin were confirmed using degumming experiments. For the first time, a scanning electron microscope energy spectrometry system was used to characterize the metal elements in the cross-section of silk and cocoons. These findings have deepened our understanding of the relationship between the overall metal ion environment and silk fiber formation and help us further conceptualize the utilization of metal ions as targets to improve the mechanical properties of the silk fibers.

## 1. Introduction

Silk fiber is one of the first natural protein fiber materials used by humans and is synthesized and secreted by the *Lepidoptera* silkworm, which has become an economically important insect [1,2]. Silk is a protein fiber material of great interest in both biological and industrial science [3,4,5]. Silk fibers comprise three protein components: fibroin, sericin, and other small molecular proteins [1]. Fibroin can be further divided into silk heavy-chain protein FibH, silk light-chain protein FibL, P25 protein, and P25-like protein [6]. The transformation of soluble animal silk protein to yield solid silk fiber is the result of a complex interplay of biochemical and physical processes [7,8]. The fibrillation of silk proteins is a complex process that involves a variety of endogenous and exogenous factors [9]. Metal ions induce conformational transitions in the silk proteins [10]. The silk glands and silk proteins of silkworms and spiders contain various metal elements, including potassium, sodium, calcium, copper, manganese, magnesium, zinc, and iron [11]. Furthermore, metal ions are highly effective in altering the structure and properties of the silk proteins [12]. Ca^2+^ can induce silk protein to form a stable protein network structure in vitro [13], and Cu^2+^ can induce the conformational transformation of silk protein solution into β-sheet crystallization [10,14]. Fe^3+^ can induce a change in the conformation of regenerated silk protein solution from a helix to a β-sheet, whereas Fe^2+^ cannot [15]. Na^+^ prevents silk protein from fibrillating in advance and maintains silk protein in a dissolved state during storage; a high concentration of K^+^ may stabilize silk fibroin’s intermolecular hydrogen bond network [16]. Moreover, the overexpression of metal ion transport-related genes (such as SERCA) can improve the mechanical properties of the silk fibers [17]. In addition, metal ions are crucial for the in vitro artificial spinning process. Superstrong silk fibers [18] were spun using a metal ion bath containing Fe^3+^ and Zn^2+^. However, previous studies have mainly focused on individual ions without analyzing the dynamic changes in the overall metal ion environment between the silk glands and silk fibers. In our previous studies, we found that the overall metal ion environment has a substantial impact on the structural transformation of the silk proteins and the properties of the silk fibers [19]. Therefore, this study aimed to analyze the dynamic change in the characteristics of metal ions in the silk glands and silk fibers of silkworms.

In this study, we aimed to systematically test the metal element content of different segments of silk glands, silk fibers, silk glands before and after spinning, different cocoon layers, fibroin, and sericin. Furthermore, we analyzed the transcription of a few metal-ion-related genes. In addition, we performed an elemental localization analysis of the cocoon layers and silk fibers using a scanning electron microscope with an energy spectrometry coupling system, for the first time. We also investigated the effects of various metal ions on silk glue proteins. Finally, an overall analysis summarizes the effects of metal ions on the conformational transitions of silk proteins. This can facilitate more effective use of metal ions for the design and modification of silk fibers.

## 2. Results

### 2.1. Distribution of Metal Ions in the Silk Glands

**Figure 1:**
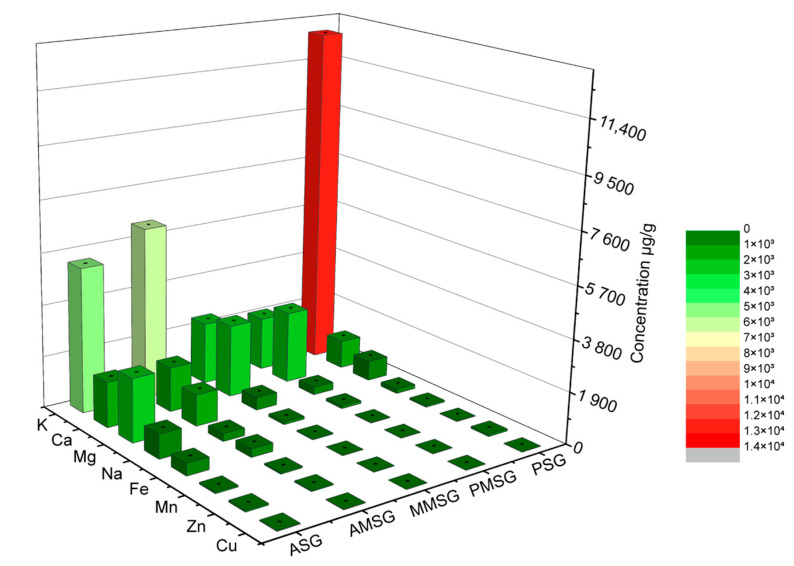
Distribution of metal ions in silk glands during the wandering stage. (ASG: anterior silk glands; AMSG: anterior part of middle silk glands; MMSG: middle part of middle silk glands; PMSG: posterior part of middle silk glands; PSG: posterior silk glands. Error bars show SD.)

Silk glands include the posterior silk gland (PSG), middle silk gland (MSG), and anterior silk gland (ASG). Each of these silk glands differs from one another in their structure and function but works in combination to complete the synthesis, secretion, and transportation of the silk proteins and the formation of the silk fibers. To obtain a comprehensive picture of the content and distribution of metal elements in these silk glands during the wandering stage, we measured the content of metal elements in different sections of each of the three silk glands using ICP-AAS (Figure 1). The data showed that Na, K, Cu, Fe, Ca, Zn, Mn, and Mg were mainly present in the silk glands, and most of the metal elements were concentrated in the anterior and middle silk glands. We also found that Na, K, and Fe were the most abundant in the anterior silk gland. Overall, silk glands contain several metallic elements, and the content of each element varies among the silk glands and different sections of each silk gland.

### 2.2. Metal Ions in the Silk Glands before and after Spinning and in the Cocoon

**Figure 2:**
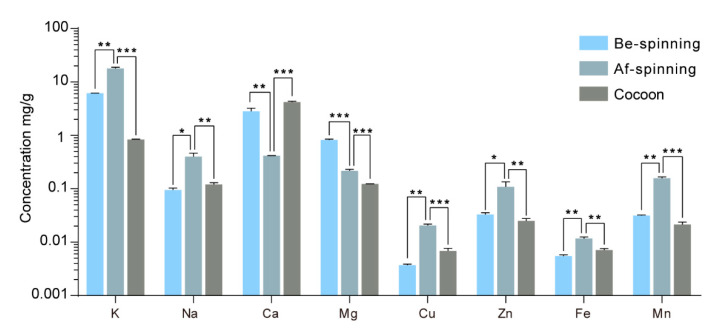
Content of metal elements in silk glands before and after spinning. (Be-spinning: before spinning, Af-spinning: after spinning. Error bars show SD, * *p* < 0.05, ** *p* < 0.01, *** *p* < 0.001 (One-way ANOVA). The y-axis data were processed by log 10).

We compared the dynamic changes in the contents of various metal elements in the silk glands before and after spinning. Silk glands before and after spinning, as well as silkworm cocoons after spinning, were selected for elemental analysis using the ICP-AAS method. We observed an apparent increase in the metal content in the silk gland before silk spinning and a decrease after spinning (Figure 2). The contents of K, Na, Cu, Zn, Fe, and Mn in the silk gland increased significantly after the completion of spinning, whereas the Ca and Mg contents significantly decreased. Regarding silkworm cocoons, only the Ca content increased significantly in comparison with metal content in silk glands after spinning, while the content of all other elements decreased.

### 2.3. Distribution of Metal Ions in Silk Fiber, Raw Silk, and Cocoon Silk

**Figure 3:**
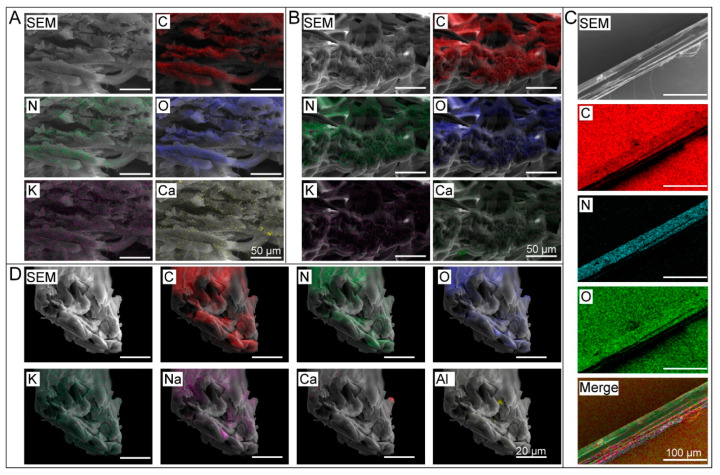
Analysis of metal elements in the cocoon layer and silk fiber. (**A**) Distribution of elements in a cross-section of cocoons of *872* strains, (**B**) Distribution of elements in a cross-section of cocoons of *Bombyx mandarina*; (**C**) Distribution of elements in a cross-section of raw silk; (**D**) Longitudinal element distribution of the silk fiber.

We analyzed the in situ distribution of metal elements in the silk fibers and cocoon layers of the silkworm strains *21-872* and *B. mandarina (Beibei)* using scanning electron microscopy coupled with energy spectroscopy. The results are shown in Figure 3. We found that K, Na, Ca, and other metal elements in high abundance could be directly observed in a cross-section of cocoon silk, whereas elements in low abundance were not detectable (Figure 3A,B). In the analysis of the longitudinal section of the silk fiber, we observed only the most basic elements (Figure 3C). We then analyzed the cross-section of raw silk, and the results showed that K, Na, Ca, and other elements were also directly observed (Figure 3D). We speculated that other elements were not detectable because of their low content. For the first time, we have clarified the spatial positions of some metal ions in silk, directly proving the existence of metal elements in cocoon silk.

### 2.4. Metal Ions in Different Cocoon Layers

**Figure 4:**
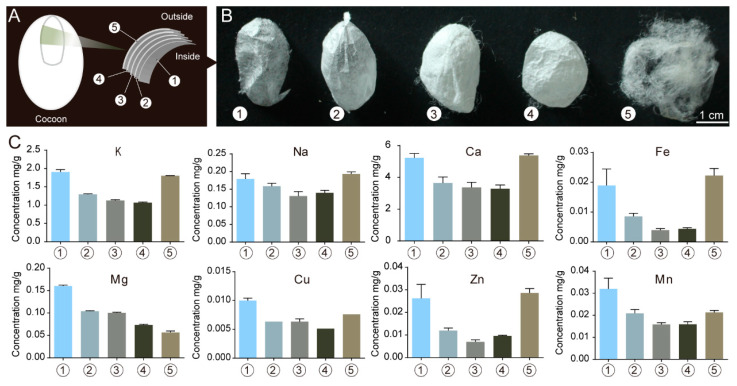
Content of metal elements in different cocoon layers of silkworm cocoons. (**A**) Schematic diagram of cocoon layers; (**B**) Photographs of different cocoon layers; (**C**) Contents of different metal elements in different cocoon layers. 1–5: From inside to outside, different cocoon layers, Error bars show SD.

In addition, we analyzed the content of metal elements in different layers of silkworm cocoons by ICP-AAS. For the analysis, the cocoons were divided into five layers from the outside to the inside; the first layer was the outermost layer and the fifth layer was the innermost layer (Figure 4A). The results of the analyses are shown in Figure 4B. The content of metal elements varied across the different cocoon layers, with the outermost layer generally having a higher content than the innermost layer. A silkworm cocoon is woven with silk fibers that are approximately 800 m long. The fibers are long and continuously formed by the silkworms over 2–3 days. Different cocoon layers are composed of fibers having different lengths but still forming a single silk fiber. The above results show that during the silk fiber formation process, the silk glands can maintain a specific metal ion environment and also dynamically adjust this environment according to the spinning stage.

### 2.5. Distribution of Metal Ions in Silk Fibroin and Sericin

**Figure 5:**
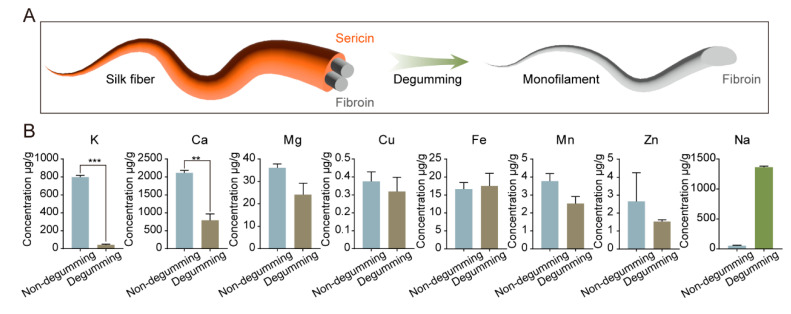
Content of metallic elements in non-degummed and fully degummed silkworm silk. (**A**): Schematic diagram of degumming; (**B**): Content changes in different metal elements before and after degumming; error bars show SD, ** *p* < 0.01, *** *p* < 0.001 (*t*-tests).

To determine the distribution of metal elements in silk fibroin and sericin, silk was completely degummed using a NaHCO_3_ solution (Figure 5A). The results of the analyses for metal element contents using ICP-AAS are shown in Figure 5B. The effect of the presence of sericin on the content of metal elements was established by comparing non-degummed silk and fully degummed silk. The content of metal elements, especially the elements K and Ca, in the completely degummed silk changed, indicating that sericin contains a high content of them (Figure 5). Conversely, the rest of the metal elements mainly existed in silk fibroin (i.e., their content in sericin is very low). The change in the Na content could not be determined due to the use of NaHCO_3_ for degumming treatment.

### 2.6. Effect of Metal Ions on the Conformation of Sericin

**Figure 6:**
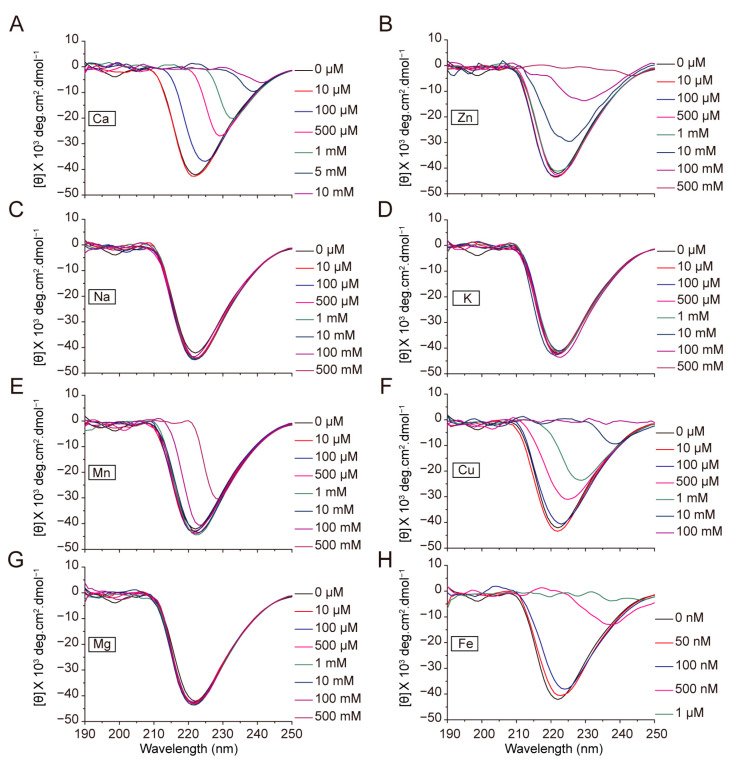
Effect of metal ions on the conformation of RS. (**A**–**H**): Circular dichroism spectra of 0.25 mg/mL regenerated sericin (RS) solution with different concentrations of metal ions.

Sericin is present in the outer layer of silk fibroin, and previous studies have focused on the effects of metal ions on silk fibroin [11,15,16,20,21]. In the middle and anterior silk gland regions, metal ions must pass through the sericin layer to reach silk fibroin; however, the effects of metal ions on sericin remain unclear. Therefore, we analyzed the effect of different metal ions on the conformation of sericin using RS solution and CD measurements. First, we decided the appropriate concentration of RS solution for the analysis to be 0.25 mg/mL. We found that the secondary structure of sericin was relatively stable at this concentration, indicating a random coil state. We then analyzed the effects of different metal ions added to the RS solution. The results showed that K, Na, Al, and Mg had no significant effect on the conformation of sericin, whereas Ca, Zn, Cu, Fe, and Mn exhibited significant effects on its conformation (Figure 6). This indicates that metal ions in the process of entering silk fibroin and some other metal ions affect the conformational transformation of sericin around the silk fiber. We speculate that the interaction between metal ions and sericin further accelerates the fibrosis of the inner silk fibroin.

### 2.7. Metal Ion-Related Genes in the Anterior Silk Gland

**Figure 7:**
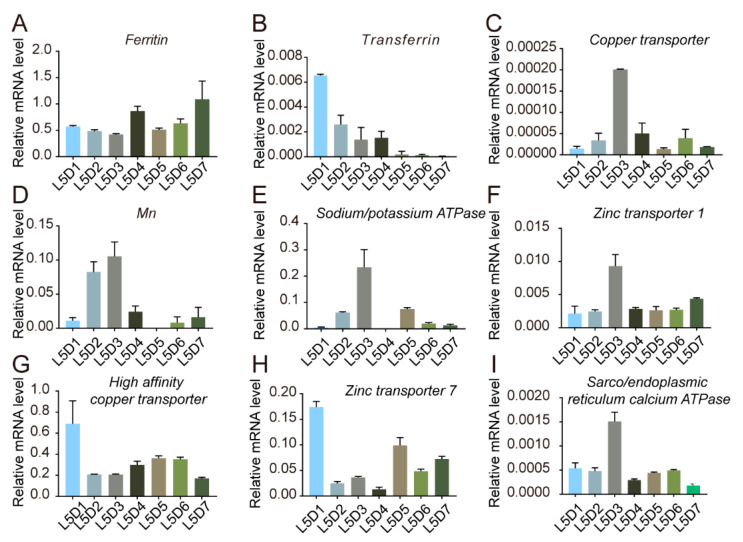
Expression profiles of metal ion-related genes in the anterior silk gland of the fifth instar larvae. (**A**–**I**) Transcript levels of different metal ion-related genes; L: larva; D: day; error bars show SD.

Metal ions are typically transported and stored by particular proteins, based on the results of studies related to anterior silk gland transcriptomics and proteomics [22]. We analyzed the expression of certain metal ion transporter-related protein genes in the anterior silk gland during the fifth instar period. The results showed that the genes in the anterior silk gland responsible for encoding these proteins had stable and active transcription levels in the fifth instar. We observed different expression patterns (Figure 7); *Copper transporter, Mn, Sodium/potassium ATPase, Sarco/endoplasmic reticulum calcium ATPase,* and *Zinc transporter 1* exhibited higher transcription levels on the third day of the fifth instar. Combined with the results of the metal element content analyses in the various silk glands and their different segments, we believe that silk glands maintain a specific metal ion environment to enable the storage and transport of the silk proteins.

## 3. Discussion

Silk fiber, a natural polymer protein material, has comprehensive mechanical properties, which are mainly reflected in the toughness of silk, but its strength and Young’s modulus are inferior to those of spider silk and other synthetic fibers (such as Kay Flask and carbon fiber) [4]. To realize diversification in silk fiber applications, it is necessary to improve its mechanical properties. Many methods have been attempted to improve the performance of silk fibers, including in vitro artificial spinning [18] and various treatments [17,23,24], such as transgenic [25,26], adding graphene or carbon nanotubes [27], strong pulling, and re-stretching processes [28,29]. However, these methods are not only time-consuming and costly but also difficult to adapt to the requirements of large-scale industrial applications. Metal ions have received considerable attention in protein-based biomaterials because they endow materials with toughness, stiffness, and even intrinsic self-healing properties [30,31]. Previous studies have confirmed that metal ions can change the conformational transition and mechanical properties of silk proteins [11,15,16,17]. In addition, a stable transgenic system was established in the silkworms [32]; therefore, metal ions can be expected to become excellent targets for regulating and designing silk fiber properties in the future.

**Figure 8:**
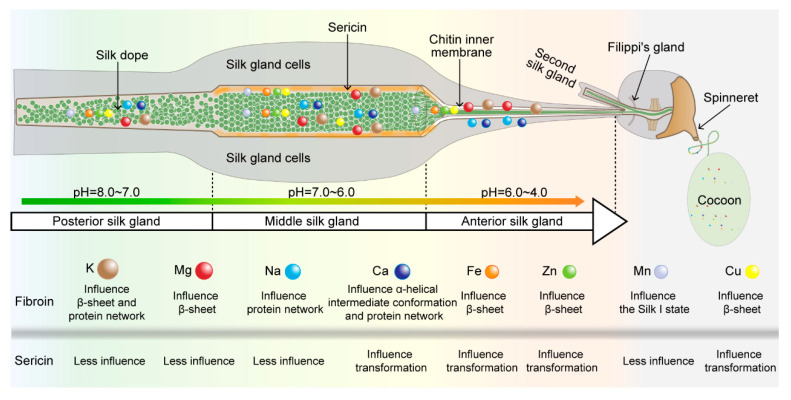
Overview diagram of metal ions and silk protein.

In our previous studies, we found that the overall metal-ion environment directly affects the structure and properties of the silk [19]. In the present study, we comprehensively analyzed the dynamic characteristics of metal ions in the silk glands and silk fibers of silkworms. From this, we confirmed that a variety of metal elements are present in the silk glands, their contents varied among different segments of silk glands, and the concentration of various metal ions increased or decreased along the silk gland duct. The anterior silk gland had the highest concentration of various metal ions and might be used as a key area for regulating metal ions. In addition, varying single metal ions, such as K^+^ and Cu^2+^, can improve the mechanical properties of the silk fibers [16]. Different metal ions have differing effects on the silk proteins but their effects on the mechanical properties of the silk fibers are still unclear. Transgenic silkworm strains can produce silk fibers that have altered mechanical properties, owing to overexpression of the sodium–potassium ion transporter (*Bm*NKA) and calcium ion transporter (*Bm*SERCA) in the spinning duct [17]. This suggests a feasible method for improving the properties of the silk fibers in an equivalent manner, but with a focus on other metal ions. Therefore, studies should analyze the role of trace metal elements. Although we cannot detect their distribution in the spatial position of the silk, they may have unexpected effects on the mechanical properties of the silk. For example, iron ion injection and in vitro ion bath experiments have found that they have a significant effect on the structure and properties of silk fibers [15,18]. In addition, the effect of metal ions on sericin cannot be ignored. Based on the existing literature [10,13,14,15,21,33], we summarized the comparison of the effects of metal ions on sericin and fibroin (Figure 8). Metal ions act mainly on silk fibroin, which is consistent with our observation that metal elements are mainly present in silk fibroin after degumming. However, our results indicate that certain metal ions are present in sericin, with four metal ions, in particular, affecting the conformational transition of sericin. Silk fibroin is synthesized in the posterior silk gland, whereas sericin is synthesized in the middle silk gland. We found that these elements had a higher content in the middle silk gland. We speculate that metal ions, along with the synthesis of silk fibroin and sericin, are dynamically adjusted to play a role in the formation of silk fibers in combination with silk proteins. The avenues of future research include co-regulating the changes in the content of metal ions affecting the silk fibroin and sericin in the middle silk gland and the anterior silk gland or designing for a complete avoidance of certain metal ions affecting the gelatinous state during the process of improving silk fibers. Finally, we found differences in the metal ion content in different layers of silkworm cocoons. The different cocoon layers correspond to different spinning stages. The silk fibers of the different cocoon segments exhibit different mechanical properties [34]. We found that the contents of various metals in the middle cocoon layer were relatively stable. As shown in Figure 8, metal ions have different effects on the conformational changes in sericin and silk fibroin. Based on the current understanding of the mechanism of silk formation, sericin and silk fibroin need to be kept in a soluble state before being spit out of the silkworm to become solidified silk fibers [35,36,37,38]. We believe that the difference in the content of metal ions in different sections of the silk gland helps to maintain the conformation of the silk proteins in each section of the silk gland and achieves the best effect when the silk protein conformation changes and silk fiber formation occurs. Thus, differences in the content of metallic elements in the various segments of the silk gland occur. We speculate that the silk glands dynamically adjust the metal element content in each spinning stage to maximize silk performance. This suggests that there is a joint regulatory mechanism for various metal ions in the silk glands. This study and its results provide promising insights for possible future strategies aimed at improving the structure of silk protein and the performance of silk fibers, intelligently and dynamically, by manipulation of the metal-ion environment of the silk glands.

## 4. Materials and Methods

### 4.1. Strain and Rearing of Silkworms and Preparation of Samples

The silkworm strains *21-872* and *Bombyx mandarina (Beibei)* were provided by the Biological Science Research Center, Academy for Advanced Interdisciplinary Studies, Southwest University. The larvae were reared under laboratory conditions (25 °C, 60–75% RH) and fed mulberry leaves until spinning. The fifth instar larvae were dissected. The other silkworms were allowed to spin cocoons naturally for subsequent analyses.

### 4.2. Degumming and Preparation of Regenerated Sericin (RS) Solution

Clean cocoons were boiled in 0.5% (*w*/*v*) NaHCO_3_ solution for 30 min, repeated three more times, and degummed thoroughly. The degummed silk fibers were dried for subsequent analyses. Silkworm cocoon fragments (1 g cocoon/30 mL water) were processed using a pressure cooker at 121 °C for 30 min. The silk fibroin was removed and the remaining solution was centrifuged (12,000 rpm) to remove impurities. The supernatant RS solution was lyophilized and stored at –20 °C. Regenerated sericin (RS) solution was used to analyze the effect of metal ions on the conformational transition of sericin. For CD testing, sericin powder was dissolved in boiling water for 20 min.

### 4.3. Element Analysis

All samples (including silk glands, silk fiber, and cocoons from the same batch) were dried in an oven overnight at 80 °C, whereafter 0.25 g of each sample was dissolved in a mixed acid (HNO_3_:HClO_4_ = 5:1), nitrified at 140 °C for 2 h, and the final solution was diluted to 50 mL with deionized water. The metal element content in the samples was measured using a Z-5000 inductively coupled plasma atomic absorption spectrometer (ICP-AAS) (Hitachi, Tokyo, Japan) using nitrous oxide-acetylene atomic absorption spectrometry. ICP-AAS was used to analyze the content of metal ions in the silk gland, sericin, silk fibroin, and different cocoon layers. All samples were tested in triplicate.

### 4.4. Scanning Electron Microscopy (SEM) and Energy Dispersive X-ray Spectroscopy (EDS) (SEM/EDS) Mapping

To analyze the in situ elemental composition of the samples (from the same batch of cocoons and silk fibers), SEM/EDS mapping tests were performed using a field-emission environmental scanning electron microscope fitted with an X-ray spectroscope (ThermoFisher Scientific, Waltham, MA, USA). The test samples were fixed onto the observation platform. An image of the sample area of interest was captured using the scanning electron microscope to perform regional positioning. Thereafter, the X-ray detector was used to obtain the distribution of the elements from the resultant image of the sample.

### 4.5. Circular Dichroism (CD) Analysis

RS solution was prepared as described above, and CD spectra of the RS were recorded using a MOS-500 CD spectrophotometer (Biologic, Seyssinet-Pariset, France) with a scanning range of 190–250 nm. The analyses were repeated independently three times. Data analysis was performed using Bio-Kine Software (version 4.74) (Biologic, Seyssinet-Pariset, France) and OriginPro Software (version 9.0) (OriginLab, Northampton, MA, USA). CD was used to analyze the effect of different metal ions on sericin conformational transition.

### 4.6. Real-Time Quantitative PCR (qPCR)

Dissecting the same batch of silkworms from the first day of the fifth instar to the spinning stage, and taking ASG for qPCR analysis. Real-time quantitative PCR was performed in three steps. First, the total RNA of the experimental samples was extracted using the TRIzol reagent (ThermoFisher Scientific). Subsequently, reverse transcription (RT) was performed using the TransScript^®^ One-Step gDNA Removal and cDNA Synthesis SuperMix (TransGen Biotech, Beijing, China), following the manufacturer’s protocol for priming with the Anchored Oligo (dT)_18_ primer. The cDNA samples were diluted to 1:10 with water. Finally, expression analyses were performed on the qTOWER3 G IVD platform (Analytik, Jena, Germany) using the NovoStart^®^ SYBR qPCR SuperMix Plus (Novoprotein, Suzhou, China). All qPCR reactions were performed in triplicate. The geometric mean of the most stably expressed reference gene (silkworm transcription initiation factor 4a (Tif4a)) was used as the endogenous control. Primer sequences are shown in Table S1.

### 4.7. Statistical Analysis

The numerical data are presented as mean and standard deviation (SD). For all the experiments, they were analyzed using GraphPad Prism software (version 7.0) (GraphPad, La Jolla, CA, USA). One-way analysis of variance (ANOVA) or Student’s *t*-test was performed between the means to determine the significant differences. For all statistical tests, statistical significance was defined as * *p* < 0.05, ** *p* < 0.01, and *** *p* < 0.001.

## 5. Conclusions

Metal ions are one of the most important factors in silk fiber formation. In this paper, we comprehensively analyzed the distribution of metal elements in silk glands, silkworm cocoons, and cocoon silk sections. A variety of metal elements were found in the silk gland, and the content was relatively high in the anterior silk gland. The spatial position of metal ions in silk was clarified, which directly proved the existence of metal elements in cocoon silk and that the content of different cocoon layers was different. It was found that metal elements mainly exist in silk fibroin, and sericin also contains a small number of metal elements. In addition, a variety of metal ion-related genes has stable and active transcription levels in the anterior silk gland of the fifth instar silkworm. The anterior silk gland was found to maintain a specific metal ion environment for the silk proteins. It further deepened the understanding of the relationship between metal ions and silk fibers and provided a basis for the subsequent improvement of silk fibers.

## Data Availability

All of the data used in this study have been provided in the main text and the Supplementary Materials.

## Supplementary Materials

The supporting information can be downloaded at: https://www.mdpi.com/article/10.3390/ijms24076556/s1.
